# Large in-plane vibrational and optical anisotropy in natural 2D heterostructure abramovite

**DOI:** 10.1038/s41598-022-21042-5

**Published:** 2022-10-07

**Authors:** Arindam Dasgupta, Dmitriy I. Belakovskiy, Ilya V. Chaplygin, Jie Gao, Xiaodong Yang

**Affiliations:** 1 Department of Mechanical and Aerospace Engineering, Missouri University of Science and Technology, Rolla, MO 65409 USA; 2 Fersman Mineralogical Museum, Russian Academy of Sciences, Moscow, Russia 119071; 3 Institute of Geology of Ore Deposits, Petrography, Mineralogy and Geochemistry, Russian Academy of Sciences, Moscow, Russia 119017; 4 Department of Mechanical Engineering, Stony Brook University, Stony Brook, NY 11794 USA

**Keywords:** Two-dimensional materials, Integrated optics

## Abstract

The design and formation of van der Waals (vdW) heterostructures with different two-dimensional (2D) materials provide an opportunity to create materials with extraordinary physical properties tailored toward specific applications. Mechanical exfoliation of natural vdW materials has been recognized as an effective way for producing high-quality ultrathin vdW heterostructures. Abramovite is one of such naturally occurring vdW materials, where the superlattice is composed of alternating Pb_2_BiS_3_ and SnInS_4_ 2D material lattices. The forced commensuration between the two incommensurate constituent 2D material lattices induces in-plane structural anisotropy in the formed vdW heterostructure of abramovite, even though the individual 2D material lattices are isotropic in nature. Here, we show that ultrathin layers of vdW heterostructures of abramovite can be achieved by mechanical exfoliation of the natural mineral. Furthermore, the structural anisotropy induced highly anisotropic vibrational and optical responses of abramovite thin flakes are demonstrated by angle-resolved polarized Raman scattering, linear dichroism, and polarization-dependent third-harmonic generation. Our results not only establish abramovite as a promising natural vdW material with tailored linear and nonlinear optical properties for building future anisotropic integrated photonic devices, but also provide a deeper understanding of the origin of structural, vibrational and optical anisotropy in vdW heterostructures.

## Introduction

Since the discovery of graphene, a plethora of two-dimensional (2D) materials have been explored extensively due to their unprecedented properties. While 2D materials exhibit remarkable electrical, optical, mechanical and chemical properties^1–3^, one of the most important findings is the design and formation of van der Waals (vdW) heterostructures by placing different 2D material layers on top of each other to create an altogether new material, where the atomically thin layers are bonded via weak vdW interactions^4,5^. It is observed that depending on the stacking symmetries and strategies of individual 2D material layers, the resultant vdW heterostructures emerge with extraordinary physical properties, paving the way for designing new materials and nanodevices with diverse functionalities^6–9^. Previous studies have demonstrated that vdW heterostructures can be used for various photonic and optoelectronic applications to create high-performance nanodevices tailored toward specific purposes^10–16^. Generally, vdW heterostructures are fabricated by layer-by-layer sequential chemical synthesis^17–19^ or mechanical restacking^20,21^ of individual 2D material layers, but these methods are cumbersome and subject to unwanted defects and interlayer adsorbates in the fabricated vdW heterostructures. Recently, the exfoliation of natural vdW superlattices has been demonstrated as an effective approach to create different types of high-quality ultrathin vdW heterostructures which are free of undesirable defects and adsorbates^22^. Naturally found sulfosalt minerals represent one such category of vdW superlattices, which are complex mixed-metal chalcogenides with the general formula A_m_B_n_C_p_, with A = Cu^1+^, Ag^1+^, Pb^2+^, Sn^2+^, Sn^4+^, Fe^2+^, Mn^2+^, Hg^2+^, Tl^1+^ and other metals, B = As^3+^, Sb^3+^ and Bi^3+^ in non-planar three-fold coordination and occasionally also Te^4+^, and C = S^2−^, Se^2−^ and Te^2−^^23,24^.

Abramovite is a very rare indium sulfosalt mineral with the simplified chemical formula of Pb_2_SnInBiS_7_, which was discovered in open cavities and fractures in fumarolic crusts on the Kudriavy volcano, Iturup Island, Kuril Islands, Russia^25,26^. The mineral is named in honor of the mineralogist Dmitry Vadimovich Abramov of the A.E. Fersman Mineralogical Museum, Russia. It is a product of high-temperature (600–620 °C) volcanic gases and forms tiny elongated lamellar-shaped crystals with silvery black color and metallic luster. Abramovite is the second sulfosalt mineral containing lead, indium and bismuth found after znamenskyite Pb_4_In_2_Bi_4_S_13_ precipitate from the Kudriavy volcanic gases^27^. It is a member of the mineral homologous series with incommensurate layered structures composed of alternated pseudo-tetragonal and pseudo-hexagonal 2D material layers, which also includes cylindrite Pb_3_Sn_4_FeSb_2_S_14_, lévyclaudite Pb_8_Sn_7_Cu_3_(Bi,Sb)_3_S_28_, merelaniite Mo_4_Pb_4_VSbS_15_, franckeite (Pb,Sn)_6_Sn_2_FeSb_2_S_14_, coiraite (Pb,Sn)_12.5_Sn_5_FeAs_3_S_28_, as well as lengenbachite Pb_6_(Ag,Cu)_2_As_4_S_13_ and cannizzarite Pb_46_Bi_54_(S,Se)_127_. Previously, the mechanical exfoliation of several members of this sulfosalt family has been demonstrated to produce ultrathin layers of vdW heterostructures. For example, cylindrite^28,29^ and franckeite^22,30^ have been studied for the formation of vdW heterostructures with constituent PbS-type and SnS_2_-type layers, while lengenbachite^31^ and cannizzarite^32^ have been exfoliated for creating vdW heterostructures with alternatively stacked PbS-type layer and As_2_S_3_- or Bi_2_S_3_-type layer. It has been observed that these resultant vdW heterostructures exhibit in-plane structural anisotropy caused by the lattice deformation between the incommensurate constituent isotropic 2D material layers.

The structural anisotropy in layered vdW materials offers an extra degree of freedom for manipulating their physical properties under optical or electrical excitations along different spatial directions. Recently, anisotropic vdW materials such as black phosphorus (BP), ReS_2_, GeSe, GeAs, and SiP have been explored for realizing many new functionalities which are not achievable in isotropic vdW materials, including polarization-sensitive photodetectors^33–35^, modulators, synaptic devices^36^, digital inverters^37^, anisotropic resistors^38^, and other building blocks for future integrated photonics^39^. The structural anisotropy in vdW heterostructure of franckeite induces anisotropic electrical properties, which has been harnessed for developing anisotropic resistors^40^ and photodetectors^30^. The structural anisotropy in vdW materials also leads to distinctive nonlinear optical responses, which can be harnessed in realizing optical nanodevices for optical communication and information processing^41^. For example, the nonlinear optical anisotropy in natural vdW heterostructure of lengenbachite has been utilized for achieving polarization-dependent intensity modulation of the converted third-harmonic optical vortices^31^. In addition, it is imperative to explore different types of anisotropic vdW heterostructures for achieving diverse and fascinating functionalities not available in the existing vdW materials to build future photonic and optoelectronic nanodevices.

Herein, we introduce abramovite as a unique type of natural anisotropic vdW heterostructures and demonstrate that ultrathin layers of vdW heterostructures of abramovite formed with alternating Pb_2_BiS_3_-type and SnInS_4_-type 2D material lattices can be obtained via the mechanical exfoliation of a natural abramovite mineral. Although the constituent 2D material lattices are isotropic in nature, the incommensurability between them makes the superlattice structurally anisotropic. The anisotropy in the crystal superlattice and the chemical composition of abramovite are first analyzed by using transmission electron microscopy (TEM), energy-dispersive X-ray spectroscopy (EDXS), and X-ray photoelectron spectroscopy (XPS). Next, the in-plane structural anisotropy of abramovite thin flakes is revealed by the anisotropic phonon vibrational modes measured from angle-resolved polarized Raman spectroscopy. The structural anisotropy induced linear dichroism in abramovite is further observed by using polarization-dependent absorption measurements. Furthermore, the effect of structural anisotropy on the nonlinear optical properties of the crystal is explored by characterizing polarization-dependent third-harmonic generation (THG) emission from abramovite thin flakes. Our results establish abramovite as a unique type of natural anisotropic vdW heterostructures with tailored vibrational and optical responses, which can be implemented for making future anisotropic photonic and optoelectronic nanodevices. The results also provide a deeper insight into the origin of structural, vibrational and optical anisotropy in vdW heterostructures.

## Results

### Crystal morphology and chemical composition of abramovite

**Figure 1:**
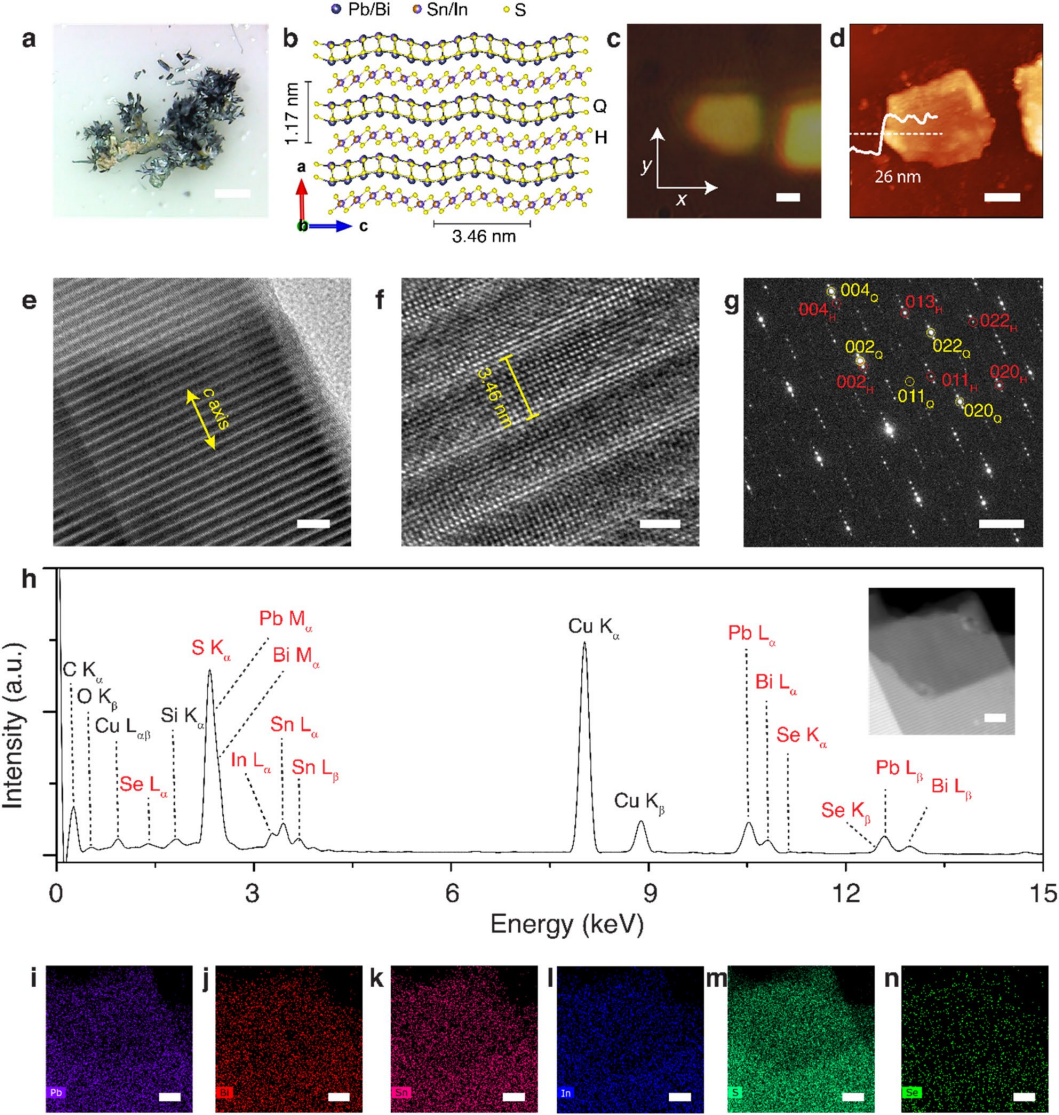
(**a**) Optical image of abramovite mineral rock, where silvery black metallic twisted and distorted lamellar crystals in several radiating aggregates are visible. Scale bar is 1 mm. (**b**) Schematic diagram of the abramovite crystal structure with vdW stacking of alternating Pb_2_BiS_3_ (Q) and SnInS_4_ (H) 2D material layers. (**c**) Reflection microscope image of one mechanically exfoliated abramovite thin flake. Scale bar is 1 µm. (**d**) AFM image of the same flake confirming the thickness of 26 nm. Scale bar is 1 µm. (**e**) TEM image of an exfoliated abramovite flake with the characteristic rippling fringes along the *c*-axis of the crystal. Scale bar is 10 nm. (**f**) Magnified HRTEM image showing the atomic arrangements. Scale bar is 2 nm. (**g**) Corresponding SAED pattern with diffraction spots related to Q-layer (yellow color) and H-layer (red color). Sale bar is 2 nm ^−1^. (**h**) Averaged EDXS spectrum from the abramovite crystal shown in the dark-field TEM image in the inset. (**i**–**n**) Corresponding TEM-EDXS elemental maps of the main elements Pb, Bi, Sn, In, S, and Se. Scale bars are 20 nm.

Figure 1a shows an image of abramovite mineral, where silvery black metallic twisted and distorted lamellar crystals in several radiating aggregates are displayed. Figure 1b shows the schematic of the simplified crystal structure of abramovite, possessing doubly incommensurate layered structures composed of alternated pseudo-tetragonal (Q) layer of Pb_2_BiS_3_ which is two atomic planes thick, and pseudo-hexagonal (H) layer of SnInS_4_ which is single-octahedral three atomic planes thick. The Q-layer contains Pb and Bi, whereas Sn and In are concentrated in the H-layer. Abramovite exhibits a triclinic crystal structure in P $\overline{1}$ space group, where the Q-layer has the lattice parameters of $a_{Q} = 23.4$ Å, $b_{{\text{Q}}} = 5.77$ Å, $c_{{\text{Q}}} = 5.83$Å, $\alpha_{{\text{Q}}} = 89.1^\circ$, $\beta_{{\text{Q}}} = 89.9^\circ$, and $\gamma_{{\text{Q}}} = 91.5^\circ$, whereas the H-layer has $a_{{\text{H}}} = 23.6$ Å, $b_{{\text{H}}} = 3.6$ Å, $c_{{\text{H}}} = 6.2$ Å, $\alpha_{{\text{H}}} = 91^\circ$, $\beta_{{\text{H}}} = 92^\circ$, and $\gamma_{{\text{H}}} = 90^\circ$^25^. It is noted that two Q–H pairs are included in the unit cell and the layer-stacking period for each Q–H pair is $11.7$Å along the *a-*axis^25^. It is indicated that $b_{{\text{Q}}}$ and $b_{{\text{H}}}$ are in an incommensurate ratio with $5b_{{\text{Q}}} = 8b_{{\text{H}}}$, while $c_{{\text{Q}}}$ and $c_{{\text{H}}}$ are in a semi-commensurate match with $12c_{{\text{Q}}} = 11c_{{\text{H}}}$ according to the vernier principle.

Abramovite thin flakes of different thicknesses are obtained by mechanically exfoliating the natural mineral using Nitto tape (SPV 224), and then the exfoliated flakes are transferred onto quartz substrate. Figure 1c is the reflection microscope image of one such mechanically exfoliated thin flake of abramovite. The corresponding atomic force microscopy (AFM) image in Fig. 1d shows the surface topography of the abramovite flake, where the height profile confirms the averaged flake thickness of 26 $\pm$ 2 nm, indicating only around 22 Q–H layer pairs. It is noted that the surface roughness of the flake is attributed to the mechanical exfoliation process during the sample preparation. The crystal structure of the exfoliated abramovite flakes is examined by TEM analysis, where the flakes are transferred on a TEM copper grid using a heat release tape. Figure 1e presents the TEM image of an abramovite thin flake where in-plane fringes of periodic darker and lighter stripes are observed along the *c-*axis, which manifests a modulated out-of-plane rippling perpendicular to the flake surface. These rippling fringes are recognized as interlayer moiré patterns in vdW heterostructures, which are ascribable to the crystal lattice deformation under the periodic strain during the forced commensuration between the incommensurate constituent Q- and H-layers. In previous studies, such rippling fringes have been noticed in other natural vdW heterostructures of sulfosalts including cylindrite^28^, franckeite^22,40^, lengenbachite^31^, and cannizzarite^32^. The atomic arrangement of the constituent lattices at the crystal surface is shown in the high-resolution (HR) TEM image in Fig. 1f. The measured period of the rippling fringes is $3.46$ nm, which is approximately half of the lattice constant *c* = 6.9 nm of the supercell, manifesting the subcell match of 12Q/11H for the abramovite crystal. The long-range match between Q and H subcells is related to the semi-commensurability along the modulation direction, according to the vernier principle with $m \cdot c_{{\text{Q}}}$ matching $n \cdot c_{{\text{H}}}$, as shown by various matches from other natural 2D misfit sulfosalts^42^. *m* and *n* are more often two successive integers with $m = n + 1$ and *n* from 10 to 15. For instance, cylindrite and lévyclaudite exhibit the 13Q/12H match. In franckeite, the match varies from 12Q/11H up to 16Q/15H as the Sn^2+^ concentration for Pb substitution is reduced. In lengenbachite, the subcell match is 12Q/11H. The match in cannizzarite is typically 12Q/7H but it changes with the variation in Pb:Bi ratio and cation:anion ratio in the crystal. While for merelaniite, the match has a 13Q/14H ratio due to the small H pseudo-layer because of the presence of Mo atoms^43^. The selected area electron diffraction (SAED) pattern along the surface normal to the [100] crystal zone axis is displayed in Fig. 1g, where the reflections corresponding to the Q-layer and H-layer can be identified distinctly. There are also multiple sets of superlattice spots presented along the [001] direction, which is an additional confirmation of the fact that the vdW heterostructure of abramovite consists of the stacked incommensurate Q-layer and H-layer.

The chemical composition of abramovite crystal is further analyzed by EDXS. Figure 1h depicts an averaged EDXS spectrum acquired from the exfoliated flake as shown in the dark-field TEM image in the inset. All the main elements of Pb, Bi, Sn, In, and S are clearly present in the crystal. In addition, a slight amount of Se is found to be present in the crystal. The presence of a small amount of Se has previously been observed in other sulfosalts^32^. The signal of Cu is present owing to the copper TEM grid. Additional peaks from other elements of C, O, and Si may be attributed to the fact that the flakes are exfoliated from the natural mineral, as well as the existing carbon film support on the TEM grid and the accumulated carbon-based adsorbates in the time of flake transfer. The compositional stoichiometry of abramovite crystal is obtained by the quantification of each element as listed in Table 1, which indicates the approximate chemical formula of Pb_2.39_Sn_1.30_In_0.83_Bi_1.02_(S_6.79_Se_0.21_)_7.00_. Although this chemical formula is close to the previously reported one for abramovite, it is not fully charge balanced which may be attributed to the low sensitivity of EDXS measurement, as well as the spectrum overlaps between S K_α_, Pb M_α_, and Bi M_α_ peaks, In L_α_ and Sn L_α_ peaks, Pb L_β_ and Se K_β_ peaks. The TEM-EDXS elemental maps of Pb, Bi, Sn, In, S, and Se are illustrated in Fig. 1i–n, which are recorded from the crystal shown in the inset of Fig. 1h. The elemental maps suggest that each main element is homogeneously distributed in the vdW heterostructure of abramovite when viewed along the *a-*axis of the crystal.

**Table 1:** EDXS quantification of elements in abramovite.

Element	Average concentration (at%)
Pb	19.09±1.92
Bi	8.09±0.83
Sn	10.37±1.07
In	6.67±0.69
S	54.07±1.75
Se	1.71±0.12

**Figure 2:**
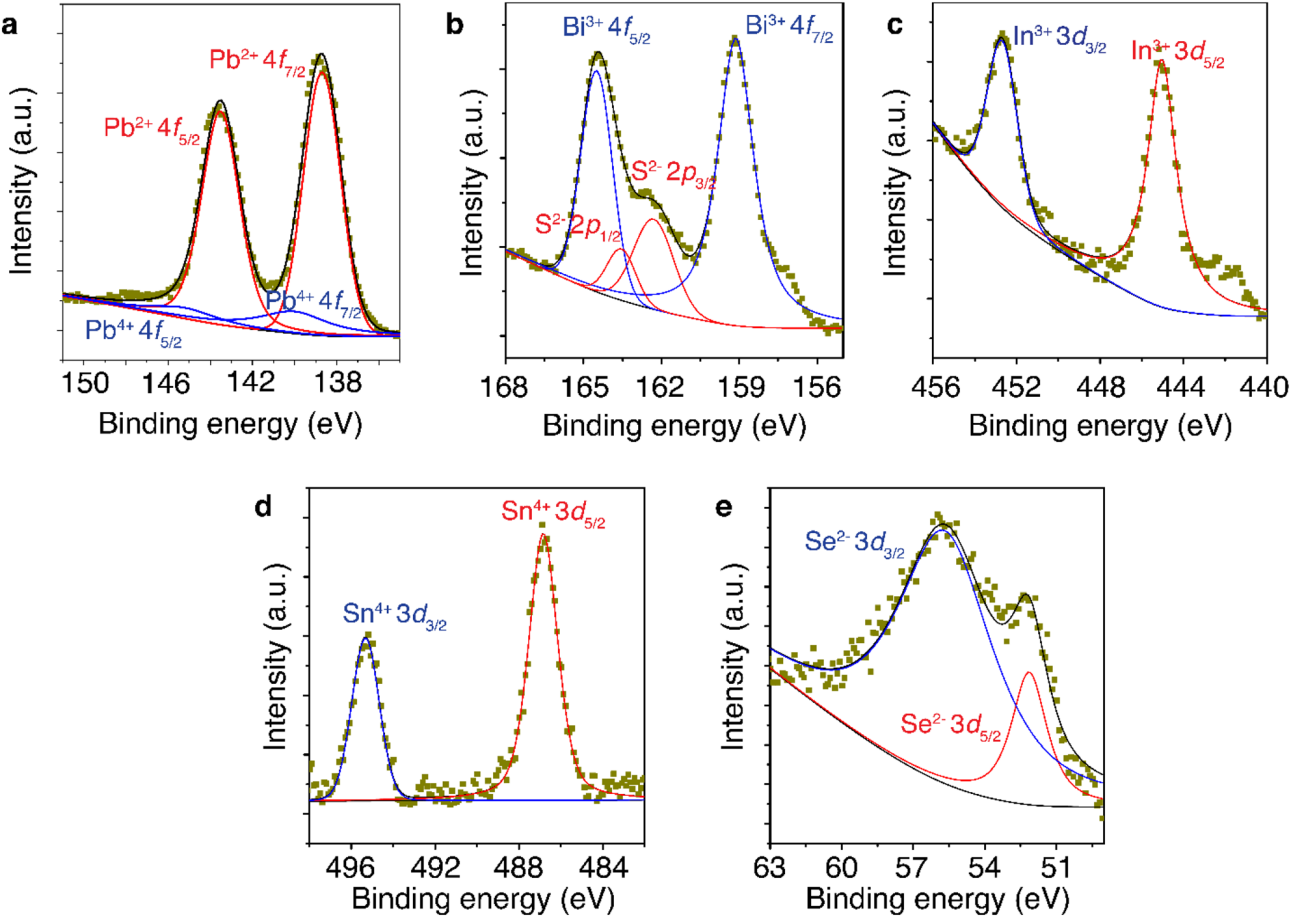
High-resolution XPS spectra around the binding energy regions of main elements in abramovite (**a**) Pb 4*f*, (**b**) Bi 4*f* and S 2*p*, (**c**) In 3*d*, (**d**) Sn 3*d*, and (**e**) Se 3*d*.

The oxidation state of each main element in abramovite crystal is then determined by XPS measurement. Figure 2a–e depict the recorded high-resolution XPS spectra around the binding energy regions of Pb 4*f*, Bi 4*f*, In 3*d*, Sn 3*d*, S 2*p*, and Se 3*d* with the corresponding peak fittings. It is shown from Fig. 2a that Pb is present in the crystal at 2 + and 4 + states with 91% to 9% ratio. Both Bi and In atoms are present at 3 + state as plotted in Fig. 2b and c. Figure 2d suggests that Sn is present in the form of 4 + state. Both S and Se are present at 2 − state as displayed in Fig. 2b and e.

### Anisotropic phonon vibrational modes in abramovite

**Figure 3:**
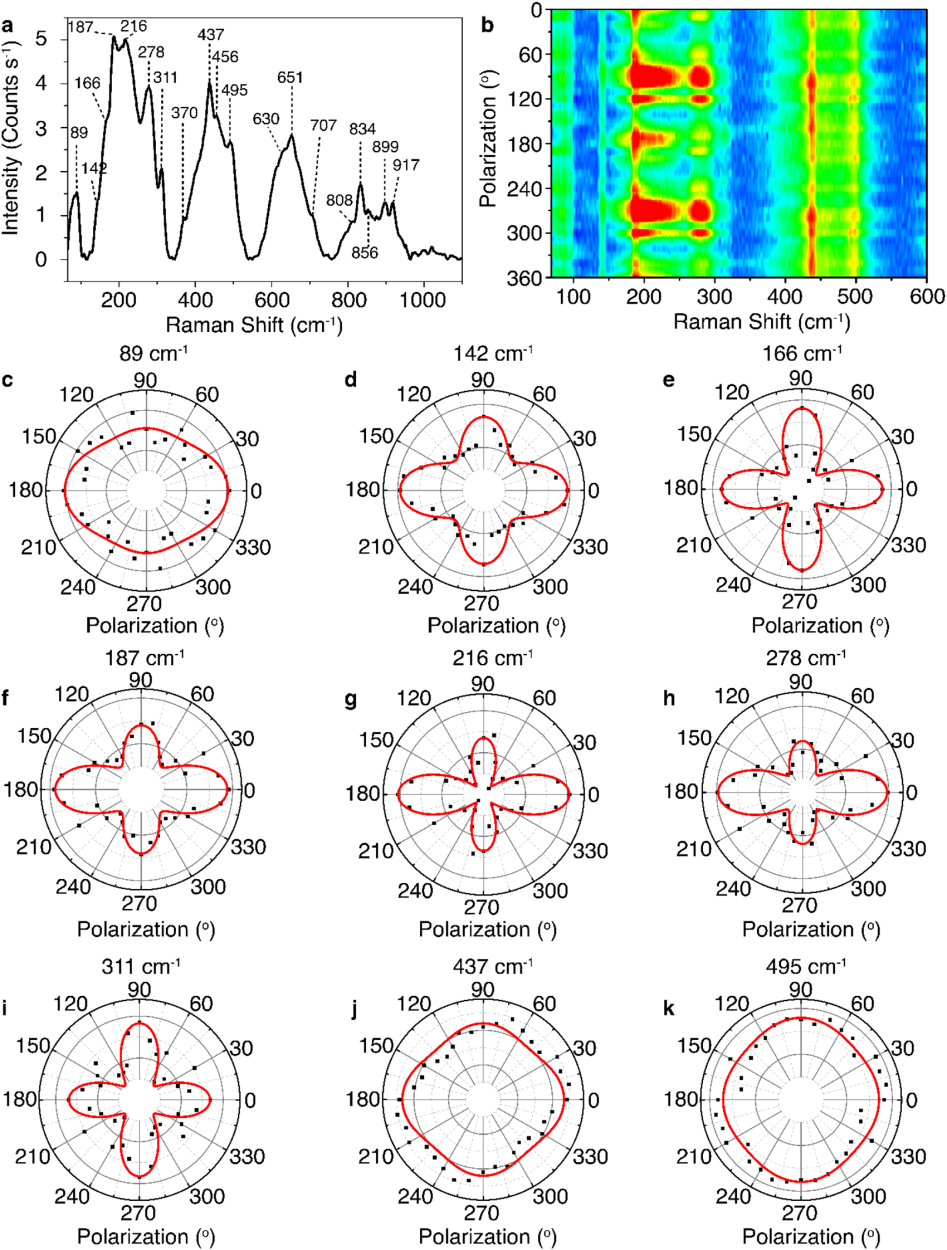
(**a**) Raman spectrum collected from the 26 nm-thick abramovite flake as shown in Fig. 1c. (**b**) Contour color map of the Raman intensity for the parallel polarization components of the Raman modes as a function of the incident linear polarization angle from the same abramovite flake. (**c**–**k**) Polar plots of the Raman intensity for the parallel polarization components of the Raman modes at 89, 142, 166, 187, 216, 278, 311, 437, and 495 cm^−1^, respectively.

Figure 3a plots the Raman spectrum measured from the 26 nm-thick abramovite flake in Fig. 1c within the spectral range of 65–1100 cm ^−1^ under the excitation of a 632.8 nm He–Ne laser. The recorded Raman spectrum exhibits multiple distinct phonon vibrational modes located at 89, 142, 166, 187, 216, 278, 311, 370, 437, 456, 495, 630, 651, 707, 808, 834, 856, 899, and 917 cm^−1^. From the XPS measurement, it is evident that the main elements in abramovite Pb, Bi, Sn, and In are all in positive oxidation states as Pb^2+^, Bi^3+^, Sn^4+^ and In^3+^, whereas S is in the S^2-^ oxidation state. Therefore, it can be inferred that the molecular bonds present in abramovite are analogous to those in the constituent binary sulfides of PbS (galena)^44,45^, Bi_2_S_3_ (bismuthinite)^46,47^, SnS_2_ (berndtite)^48,49^, In_2_S_3_ (indium sulfide)^50–52^. The 89 cm^−1^ peak is assigned as the B_1g_ vibrational mode of Bi_2_S_3_ (81 cm^−1^). The 142 cm^−1^ peak corresponds to the second-order difference spectrum of SnS_2_ lattices (141 cm^−1^), as well as the transverse acoustic and optical phonon modes of PbS lattices (154 cm^−1^). The 166 cm^−1^ peak is attributed to the B_1g_ mode of Bi_2_S_3_ (168 cm^−1^). The peak at 187 cm^−1^ represents a combination of the A_g_ mode of Bi_2_S_3_ (186 cm^-1^) and the F_2g_ vibration of In_2_S_3_ (180 cm^−1^). The 216 cm^−1^ peak is related to the *E*_*g*_ mode of SnS_2_ (205 cm^−1^) and the longitudinal optical phonon modes of PbS lattices (204 cm^-1^). The 278 cm^−1^ peak is assigned as the B_1g_ mode of Bi_2_S_3_ (277 cm^−1^) and the E_g_ vibration of In_2_S_3_ (266 cm^−1^). The peak at 311 cm^-1^ is attributed to a combination of the A_1g_ mode of SnS_2_ (315 cm^−1^) and the A_1g_ vibration of In_2_S_3_ (306 cm^−1^). The peak at 370 cm^−1^ is assigned as the A_1g_ vibrational mode of In_2_S_3_ (365 cm^−1^). The 437 and 456 cm^−1^ peaks are related to the first overtone of the longitudinal optical phonons of PbS (454 cm^−1^). The 495 cm^−1^ peak is related to the S–S bond stretching vibrational mode (480 cm^−1^). The Raman modes about 630 and 651 cm^−1^ are attributed to the second overtone of the longitudinal optical phonons of PbS (630 cm^−1^) and the second-order effects of SnS_2_ lattices (550–650 cm^−1^). Finally, the broad Raman shifts between 800 and 920 cm^−1^ may be related to the first-order and second-order phonon vibrational modes due to the resonant Raman scattering in the component sulfides. It has been previously observed in the Raman study under high laser excitation power (> 5 mW) that the laser-induced photo-oxidation of Bi_2_S_3_ and PbS will form oxysulfates of Bi_2_O_2_(SO_4_) and PbO·PbSO_4_^46,53^. Here, the characteristic 965 cm^−1^ peak corresponding to the symmetric stretching mode of sulphate ion SO_4_^2−^ is not observed in the measured Raman spectrum due to the applied low laser excitation power (< 1 mW) to avoid any potential thermal and photochemical degradation of the sample.

The structural anisotropy in a crystal gives rise to the anisotropy in phonon vibrations^54^. Hence, angle-resolved polarized Raman spectroscopy studies can unveil the in-plane structural anisotropy in the triclinic crystal of abramovite by analyzing the anisotropic phonon vibrational modes. This method has previously been used for determining the crystal anisotropy in other sulfosalt minerals such as cylindrite^28^, frankeite^40^, lengenbachite^31^, cannizzarite^32^, and nagyágite^55^. Figure 3b shows the contour color map of the variation of Raman intensity for the parallel polarization components of the Raman modes within the range of 65 to 600 cm^-1^ as a function of the incident linear polarization angle $\theta$, where $\theta =0^\circ$ for the polarization along the *x*-axis as labeled in Fig. 1c. The observed periodic variations in the intensity for all the Raman modes indicate the anisotropic responses of phonon vibrations, which can be understood in light of the classical Raman selection rule. Since abramovite has a triclinic crystal structure, it supports only nondegenerate A_g_ vibrational modes. The Raman tensor for each A_g_ vibrational mode of abramovite can be expressed as,

1 $${\mathbf{R}} = \left[ {\begin{array}{*{20}c} a & d & e \\ d & b & f \\ e & f & c \\ \end{array} } \right]$$

with *a, b, c, d, e, f* being the complex elements of the Raman tensor. The values of *a, b, c, d, e, f* are different for each Raman mode. The Raman intensity is then written as $I \propto \left| {{\mathbf{e}}_{{\text{i}}} \cdot {\mathbf{R}} \cdot {\mathbf{e}}_{{\text{s}}} } \right|$ with ${\mathbf{e}}_{{\text{i}}}$ and ${\mathbf{e}}_{{\text{s}}}$ representing the unit polarization vectors of the incident and scattered light, where $${\mathbf{e}}_{{\text{i}}} = {\mathbf{e}}_{{\text{s}}} = \left[ {\begin{array}{*{20}c} {\cos \theta } & {\sin \theta } & 0 \\ \end{array} } \right]$$ for the parallel polarization component and $\theta$ is the incident polarization angle with respect to the *x*-direction. Thus, the Raman intensity for the parallel polarization components of the ${\text{A}}_{{\text{g}}}$ vibrational modes in abramovite depending on the incident polarization angle can be expressed as^31^,

2 $$\begin{gathered} I_{\parallel } \left( \theta \right) \propto \left| a \right|^{2} \cos^{4} \theta + \left| b \right|^{2} \sin^{4} \theta + \left( {4\left| d \right|^{2} + 2\left| a \right|\left| b \right|\cos \phi_{ab} } \right)\sin^{2} \theta \cos^{2} \theta \hfill \\ \quad \quad \quad + 4\left| a \right|\left| d \right|\cos \phi_{ad} \sin \theta \cos^{3} \theta + 4\left| b \right|\left| d \right|\cos \phi_{bd} \sin^{3} \theta \cos \theta \hfill \\ \end{gathered}$$

where $\phi_{ab}$, $\phi_{ad}$, $\phi_{bd}$ are the phase differences between *a* and *b*, *a* and *d*, *b* and *d*. The polar plots of the evolution of the Raman intensity for the parallel polarization component of each A_g_ vibrational mode at 89, 142, 166, 187, 216, 278, 311, 437, and 495 cm^−1^ with the incident polarization angle are displayed in Fig. 3c–k, where the collected data in black points are fitted with Eq. (2) in red curves. It is notable that most of the phonon vibrational modes show an anisotropic two-fold symmetric pattern with a maximum at $\theta = 0^\circ$ and a secondary maximum at $\theta = 90^\circ$, whereas the vibrational modes at 311 and 495 cm^−1^ exhibit the anisotropic patterns with a maximum at $\theta = 90^\circ$ and a secondary maximum at $\theta = 0^\circ$. Previous studies on other triclinic crystals suggest that the crystal axes are orientated at the incident polarization angles where either a maximum or a secondary maximum Raman intensity is obtained for the parallel component of the A_g_ mode^28,31,56^. Thereby, it is inferred that the rippling direction (*c-*axis) of the 26 nm-thick abramovite flake shown in Fig. 1c is aligned along either $\theta = 0^\circ$ (*x-*axis) or $\theta = 90^\circ$ (*y*-axis).

### Structural anisotropy induced linear dichroism in abramovite

**Figure 4:**
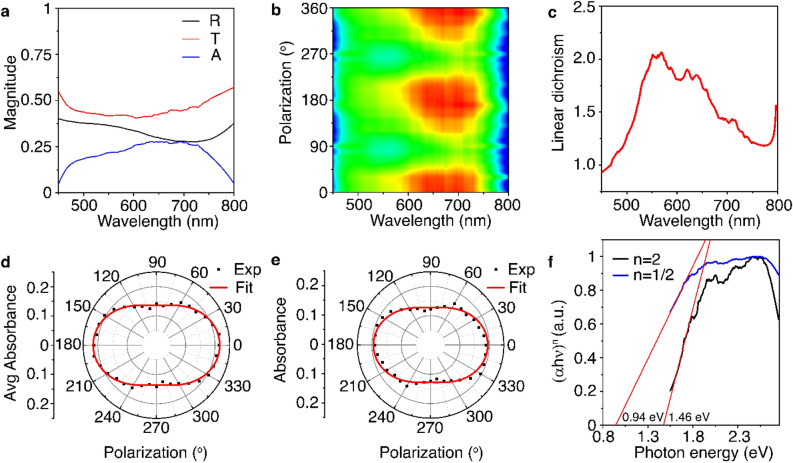
(**a**) Measured reflectance (*R*), transmittance (*T*), and absorbance (*A*) spectra from the 26 nm-thick abramovite flake. (**b**) Contour color map of the variation of absorbance spectrum as a function of the incident linear polarization angle. (**c**) $A_{x} /A_{y}$ ratio as a function of the incident wavelength. (**d**) Polar plot of the average absorbance over the measured wavelength range as a function of the incident polarization angle. (**e**) Polar plot of the absorbance at the wavelength of 520 nm as a function of the incident polarization angle. (**f**) Tauc plots for the extraction of the direct and indirect optical band gaps in abramovite crystal.

Structural anisotropy in a vdW heterostructure induces linear dichroism. Previously, polarization-dependent absorption measurements have been used to probe the linear dichroism and the crystal axes of other natural anisotropic vdW heterostructures such as cylindrite^28^, franckeite^40^, lengenbachite^31^, cannizzarite^32^, and nagyágite^55^. With that hindsight, next we investigate the evolution of the absorbance in abramovite crystal depending on the incident light polarization for the same 26 nm-thick flake. The black and red curves in Fig. 4a represent the measured reflectance (*R*) and transmittance (*T*) spectra in the wavelength range of 450–800 nm from the flake, when the incident linear polarization is along the *x-*axis ($\theta = 0^\circ$). The reflectance shows a slight dip around the wavelength of 730 nm, while the transmittance remains almost flat with a shallow and broad dip centered around 600 nm. The absorbance (*A*) spectrum calculated from *A* = 1 − *R* − *T* is plotted as the blue curve in Fig. 4a, which shows a broad peak centered near 700 nm. Figure 4b plots the contour color map of the variation of absorbance spectrum as a function of the incident linear polarization angle. The periodic variations in the absorbance at different incident polarization angles directly reveals the structural anisotropy in abramovite crystal. It is noticed that the absorbance reaches the maximum when the incident polarization angle is $\theta = 0^\circ$ and the minimum when the polarization angle is $\theta = 90^\circ$. As a consequence, it can be inferred that the crystal’s *c*-axis is oriented along the *x-*direction at $\theta = 0^\circ$. These results also indicate that abramovite crystal exhibits linear dichroism. Figure 4c shows the evolution of the dichroic ratio of linear dichroism as a function of the incident wavelength by plotting the $A_{x} /A_{y}$ ratio between the absorbance $A_{x}$ and $A_{y}$ at the incident polarization angle of $\theta = 0^\circ$ and $\theta = 90^\circ$. Within the wavelength range of 450–800 nm, the $A_{x} /A_{y}$ ratio is as high as 2.06 at 570 nm, indicating strong linear dichroism response and large in-plane optical anisotropy in abramovite crystal. The polar plot in Fig. 4d further shows the angular dependence of the average absorbance integrated over the entire wavelength range of 450–800 nm as a function of the incident linear polarization angle. A dipolar evolution pattern of the average absorbance is observed, which is fitted with a sinusoidal function in the form of $A\left( \theta \right) = A_{x} \cos^{2} \theta + A_{y} \sin^{2} \theta$, and the average $A_{x} /A_{y}$ ratio of 1.59 is obtained. Figure 4e is the polar plot of the variation of absorbance at the wavelength of 520 nm as a function of the incident polarization angle, where the $A_{x} /A_{y}$ ratio of 1.52 is attained. The observed strong linear dichroism effect reflects the structural anisotropy in abramovite crystal, where the *x*-axis and *y*-axis are identified as the rippling direction of the crystal’s *c-*axis and its perpendicular direction of the *b*-axis, respectively.

The optical band gap $E_{g}$ of abramovite crystal is further estimated by using the Tauc plot with the expression of $\left( {\alpha h\nu } \right)^{n} = m\left( {h\nu - E_{g} } \right)$, where *m* is a constant and $\alpha$ is the absorption coefficient at the photon energy $h\nu$, with the Plank’s constant $h$ and the photon frequency $\nu$. Here, $n = 1/2$ represents an allowed indirect transition, while $n = 2$ signifies an allowed direct transition. The absorption coefficient ($\alpha$) spectrum can be extracted from the reflectance (*R*) and transmittance (*T*) spectra in Fig. 4a as^57^,

3 $$\alpha = \frac{1}{d}{\text{ln}}\left\{ {\frac{2T}{{\left[ {T^{2} - \left( {1 - R} \right)^{2} } \right] + \left\{ {\left[ {T^{2} - \left( {1 - R} \right)^{2} } \right]^{2} + 4T^{2} } \right\}^{1/2} }}} \right\}$$

where *d* = 26 nm is the thickness of the abramovite flake. Figure 4f depicts the values of $\left( {\alpha h\nu } \right)^{2}$ (black curve) and $\left( {\alpha h\nu } \right)^{1/2}$ (blue curve) as a function of the photon energy ($h\nu$), showing a good linear fit in the Tauc plots for both cases. The Tauc plots indicate an allowed direct transition at 1.46 eV and an allowed indirect transition at 0.94 eV, corresponding to the direct optical band gap and the indirect optical band gap in abramovite crystal, respectively. It is noted that the estimated band gap values of abramovite is within the band gap range covered by its constituent binary sulfides of PbS at 0.37 eV, Bi_2_S_3_ at 1.3 eV, In_2_S_3_ at 2.1 eV and SnS_2_ at 2.2 eV, which agrees with the previous studies in the optical band gaps of sulfosalt minerals^58^.

### Anisotropic nonlinear optical response in abramovite

**Figure 5:**
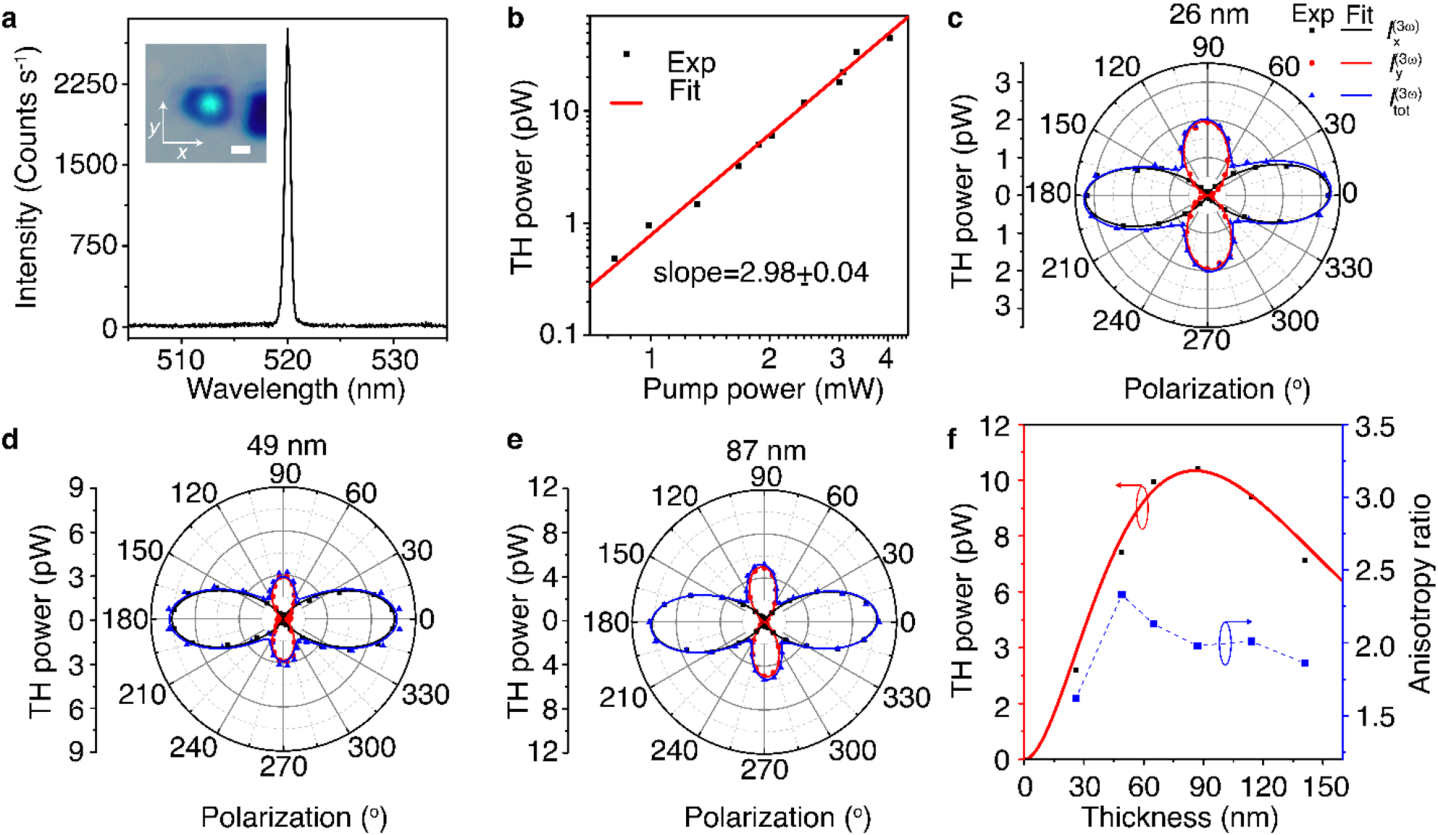
(**a**) THG emission spectrum from the 26 nm-thick abramovite flake. Inset shows the transmission microscope image of the THG emission from the illuminated area of the flake. Scale bar is 1 µm. (**b**) Log-scale plot of the measured TH power depending on the pump power. (**c**) Dependence of the TH power on the linear polarization angle of the pump beam for the 26 nm flake. (**d**,**e**) Polarization-dependent THG emission from two more flakes with thicknesses of 49 and 87 nm. (**f**) Evolution of the measured TH power (black data points) and THG anisotropy ratio (blue data points) as a function of the flake thickness. The red curve is the theoretical fit to the TH power.

Furthermore, to explore the effect of structural anisotropy on the nonlinear optical response of abramovite, polarization-dependent anisotropic THG emission from abramovite thin flakes is investigated. The inset in Fig. 5a is a transmission optical microscope image of the THG emission from the same 26 nm-thick abramovite flake, under the excitation from a pulsed laser of 1560 nm fundamental wavelength and 1.5 µm spot size. The spectral response of the THG emission in Fig. 5a shows that the emission peak is at 520 nm, which is one-third of the fundamental wavelength. Figure 5b is the log-scale plot of the average THG power depending on the average pump power, where the observed cubic power-law dependence confirms the THG process. At the average pump power of 4.03 mW which is equivalent to a peak irradiance of 27.4 GW cm^−2^, a THG conversion efficiency of $1.1\times {10}^{-8}$ is obtained for the 26 nm-thick abramovite flake. According to the observed high in-plane anisotropy in optical absorbance and linear dichroism in abramovite crystal, strong anisotropy in THG response is also expected. We consider a pump beam with linear polarization at fundamental frequency $\omega$ as ${\overrightarrow{\mathbf{E}}}^{(\omega )}={E}_{0}\widehat{p}$, where $\widehat{p}=\widehat{x}\mathrm{cos}\theta +\widehat{y}\mathrm{sin}\theta$ defines the unit polarization vector oriented along an angle $\theta$ with respect to the *x*-axis in the *x–y* plane. For the crystal axes *c*, *b*, and *a* oriented along the *x*, *y*, and *z*-directions, the contracted form of the third-order nonlinear susceptibility tensor of the triclinic abramovite crystal can be written as^28^,

4 $$\chi^{{\left( {3\omega } \right)}} = \left[ {\begin{array}{*{20}c} {\chi_{11} } \\ {\chi_{21} } \\ {\chi_{31} } \\ \end{array} \begin{array}{*{20}c} {\chi_{12} } \\ {\chi_{22} } \\ {\chi_{32} } \\ \end{array} \begin{array}{*{20}c} {\chi_{13} } \\ {\chi_{23} } \\ {\chi_{33} } \\ \end{array} \begin{array}{*{20}c} {\chi_{14} } \\ {\chi_{24} } \\ {\chi_{34} } \\ \end{array} \begin{array}{*{20}c} {\chi_{15} } \\ {\chi_{25} } \\ {\chi_{35} } \\ \end{array} \begin{array}{*{20}c} {\chi_{16} } \\ {\chi_{26} } \\ {\chi_{36} } \\ \end{array} \begin{array}{*{20}c} {\chi_{17} } \\ {\chi_{27} } \\ {\chi_{37} } \\ \end{array} \begin{array}{*{20}c} {\chi_{18} } \\ {\chi_{28} } \\ {\chi_{38} } \\ \end{array} \begin{array}{*{20}c} {\chi_{19} } \\ {\chi_{29} } \\ {\chi_{39} } \\ \end{array} \begin{array}{*{20}c} {\chi_{10} } \\ {\chi_{20} } \\ {\chi_{30} } \\ \end{array} } \right]$$

where the first subscript 1, 2, 3 refers to *x*, *y*, *z*, and the second subscript signifies the following combined components,

$$\begin{array}{*{20}c} {xxx} \\ 1 \\ \end{array} \begin{array}{*{20}c} {yyy} \\ 2 \\ \end{array} \begin{array}{*{20}c} {zzz} \\ 3 \\ \end{array} \begin{array}{*{20}c} {yzz} \\ 4 \\ \end{array} \begin{array}{*{20}c} {yyz} \\ 5 \\ \end{array} \begin{array}{*{20}c} {xzz} \\ 6 \\ \end{array} \begin{array}{*{20}c} {xxz} \\ 7 \\ \end{array} \begin{array}{*{20}c} {xyy} \\ 8 \\ \end{array} \begin{array}{*{20}c} {xxy} \\ 9 \\ \end{array} \begin{array}{*{20}c} {xyz} \\ 0 \\ \end{array}$$

Thus, the induced third-order nonlinear polarization in abramovite crystal can be written as,

5 $${\vec{\mathbf{P}}}^{{\left( {3\omega } \right)}} = \left[ {\begin{array}{*{20}c} {P_{x}^{{\left( {3\omega } \right)}} } \\ {P_{y}^{{\left( {3\omega } \right)}} } \\ {P_{z}^{{\left( {3\omega } \right)}} } \\ \end{array} } \right] = \varepsilon_{0} E_{0}^{3} \left[ {\begin{array}{*{20}c} {\chi_{11} \cos^{3} \theta + \chi_{12} \sin^{3} \theta + 3\chi_{18} \cos \theta \sin^{2} \theta + 3\chi_{19} \sin \theta \cos^{2} \theta } \\ {\chi_{21} \cos^{3} \theta + \chi_{22} \sin^{3} \theta + 3\chi_{28} \cos \theta \sin^{2} \theta + 3\chi_{29} \sin \theta \cos^{2} \theta } \\ 0 \\ \end{array} } \right]$$

Since the third-harmonic (TH) electric field can be expressed as ${\vec{\mathbf{E}}}^{{\left( {3\omega } \right)}} \propto P_{x}^{{\left( {3\omega } \right)}} \hat{x} + P_{y}^{{\left( {3\omega } \right)}} \hat{y}$, the *x-* and *y*-polarization components of the TH intensity is given by,

6 $$\begin{gathered} I_{x}^{{\left( {3\omega } \right)}} \propto \left( {\chi_{11} \cos^{3} \theta + \chi_{12} \sin^{3} \theta + 3\chi_{18} \cos \theta \sin^{2} \theta + 3\chi_{19} \sin \theta \cos^{2} \theta } \right)^{2} \hfill \\ I_{y}^{{\left( {3\omega } \right)}} \propto \left( {\chi_{21} \cos^{3} \theta + \chi_{22} \sin^{3} \theta + 3\chi_{28} \cos \theta \sin^{2} \theta + 3\chi_{29} \sin \theta \cos^{2} \theta } \right)^{2} \hfill \\ \end{gathered}$$

The polar plot in Fig. 5c shows the evolution of the TH power depending on the incident linear polarization angle $\theta$ at the pump power of 1.67 mW. The linear polarization of the pump beam is set by putting a linear polarizer along the *x*-axis (the crystal’s *c*-axis) and a rotating half-wave plate. The measured *x-*component, *y*-component, and total TH power are plotted as black, red, and blue points, while the solid curves are fitted with Eq. (6). The theoretical fits agree well with the experimental data. It is shown that the polarization-dependent THG emission pattern from the abramovite crystal exhibits an anisotropic two-fold rotational symmetry. The maximum TH power is obtained as the linear polarization angle of the pump beam is $\theta = 0^\circ$ which is along the crystal’s *c*-axis, while the second maximum occurs at $\theta = 90^\circ$ as the incident linear polarization is along the crystal’s *b*-axis. As illustrated in Fig. 5d and e, similar anisotropic THG emission patterns are also detected in the abramovite flakes with thicknesses of 49 and 87 nm. Next, the structural anisotropy of abramovite crystal is unveiled by estimating the ratio between the relative magnitudes of the $\chi^{\left( 3 \right)}$ tensor elements by using Eq. (6) for the abramovite flakes of different thicknesses. For the 26 nm-thick abramovite flake, the ratio of the $\chi^{\left( 3 \right)}$ tensor elements is $\chi_{11} :\chi_{12} :\chi_{18} :\chi_{19} :\chi_{21} :\chi_{22} :\chi_{28} :\chi_{29}$ = 1:0.015:0.073:0.022:0.007:0.781:0.004:0.147 with $\chi_{11} /\chi_{22}$ = 1.28, manifesting the intrinsic nature of anisotropic nonlinear optical response in abramovite crystal. It is noteworthy that the crystal anisotropy is slightly higher for the flakes with thicknesses of 49 and 87 nm, which have the ratios of $\chi_{11} :\chi_{12} :\chi_{18} :\chi_{19} :\chi_{21} :\chi_{22} :\chi_{28} :\chi_{29}$ = 1:0.004:0.059:0.018:0.011:0.595:0.008:0.047 and 1:0.016:0.052:0.029:0.008:0.702:0.006:0.058 with $\chi_{11} /\chi_{22}$ = 1.68 and 1.42, respectively. Further, the THG anisotropy ratio of the total TH power $I_{tot }^{{\left( {3\omega } \right)}} \left( {\theta = 0^\circ } \right)/I_{tot }^{{\left( {3\omega } \right)}} \left( {\theta = 90^\circ } \right)$ for the flakes with different thicknesses from 26 to 141 nm is plotted in Fig. 5f as the blue data points. It is observed that the THG anisotropy ratio has a slightly low value of 1.67 for the 26 nm flake, but it remains more or less close to the average value of 1.99 for other thicknesses with the highest value of 2.3 for the 49 nm flake. Additionally, the estimated average $\chi_{11} /\chi_{22}$ value is 1.41 for the abramovite flakes in the thickness range of 26 to 141 nm.

Moreover, the TH emission power as a function of the abramovite flake thickness is studied and the value of the third-order nonlinear susceptibility is estimated. The measured total TH power ($p^{{\left( {3\omega } \right)}}$) as a function of the flake thickness ($d$) at the pump power of $p^{\left( \omega \right)}$ = 1.67 mW is presented in Fig. 5f as the black data points. For this measurement, the incident linear polarization of the pump beam is kept along the *c-*axis of the corresponding flakes. The trend in the thickness dependence of the THG response is determined by two competing factors. One is the amount of the material irradiated by the pump laser and the other is the absorption of the THG emission within the material. As the flake thickness is increased, both the amount of material and the absorption increase. A quadratic increase in the TH power is observed as the flake gets thicker and a peak THG conversion efficiency of 6.23 × 10^−9^ is obtained for the 87 nm flake. Until this thickness, the absorption in the material is less dominant. Afterwards, an exponential decay in the TH power is observed as the flake thickness is increased larger than 87 nm, because at these thicknesses, the high absorption of THG emission becomes predominant. Hence, the TH power is fitted with an exponential decay function of the form $p^{{\left( {3\omega } \right)}} \left( d \right) = Cd^{2} \exp \left( { - \frac{{4{\uppi }k_{3} d}}{{\lambda_{3} }}} \right){ }$, where $C$ is a fitting parameter and $k_{3}$ is the imaginary part of the refractive index of abramovite at the TH wavelength $\lambda_{3} = 520$ nm. The theoretical fit to the TH power is plotted as the red curve in Fig. 5f, which indicates the $k_{3}$ value of 0.965 for abramovite crystal. Besides, the effective scalar value of the third-order nonlinear susceptibility $\chi_{eff}^{\left( 3 \right)}$ of abramovite crystal is estimated from the formula^59^,

7 $$\chi_{eff}^{\left( 3 \right)} = \left( {\frac{{16\sqrt {n_{3}^{2} + k_{3}^{2} } n_{1}^{3} \in_{0}^{2} c^{4} f_{rep}^{2} W^{4} \tau^{2} \left[ {\frac{\pi }{4\ln 2}} \right]^{3} p^{{\left( {3\omega } \right)}} }}{{9\omega^{2} d^{2} p^{\left( \omega \right)3} }}\left( {\frac{{\left( {\frac{{4\pi^{2} k_{3}^{2} d^{2} }}{{\lambda_{3}^{2} }} + \Delta k^{2} d^{2} } \right)}}{{e^{{ - \frac{{4\pi k_{3} d}}{{\lambda_{3} }}}} - 2\cos \left( {\Delta kd} \right)e^{{ - \frac{{2\pi k_{3} d}}{{\lambda_{3} }}}} + 1}}} \right)e^{{\frac{{4\pi k_{3} d}}{{\lambda_{3} }}}} } \right)^{\frac{1}{2}}$$

where $\in_{0}$ is the vacuum permittivity, *c* is the speed of light, $n_{1}$ and $n_{3}$ are the real part of the refractive index of abramovite at the fundamental wavelength $\lambda_{1} = 1560$ nm and TH wavelength $\lambda_{3} = 520$ nm, and ${\Delta }k = \frac{{6{\uppi }}}{{\lambda_{1} }}\left( {n_{1} - n_{3} } \right)$ is the phase mismatch between the forward propagating fundamental beam and the TH beam in the transmission microscope arrangement. The parameters of the Gaussian fundamental pulsed laser beam include pump power $p^{\left( \omega \right)}$ = 1.67 mW, beam spot size $W = 1.5$ µm, laser repetition rate $f_{rep} = 80$ MHz, and pulse width $\tau =$ 90 fs. It is worth mentioning that the refractive index information of abramovite crystal is still unavailable, but the refractive indices of its constituent binary sulfides are known, with $n_{1} =$ 4.24 and $n_{3} =$ 4.34 for PbS, $n_{1} =$ 2.4 and $n_{3} =$ 2.3 for SnS_2_^60^, $n_{1} =$ 2.4 and $n_{3} =$ 2.8 for In_2_S_3_^61^, $n_{1} =$ 1.9 and $n_{3} =$ 2.6 for Bi_2_S_3_^62^. Thereby, assuming an averaged refractive index of $n_{1} = n_{3} =$ 3.0 for abramovite crystal, the $\chi_{eff}^{\left( 3 \right)}$ value is estimated as $3.87 \times 10^{ - 19}$ m^2^ V^-2^, while as the refractive index varies from 2.0 to 4.0, the $\chi_{eff}^{\left( 3 \right)}$ value is between $1.77 \times 10^{ - 19}$ and $6.81 \times 10^{ - 19}$ m^2^ V^−2^.

Finally, the nonlinear optical response of abramovite crystal is assessed by comparing the measured $\chi^{\left( 3 \right)}$ value and $\chi_{11} /\chi_{22}$ anisotropy ratio with the previously reported values of other natural and synthetic anisotropic vdW materials, which are tabulated in Table 2. It indicates that abramovite exhibits strong nonlinear optical response and holds the highest $\chi^{\left( 3 \right)}$ value among the recently demonstrated natural anisotropic vdW materials from sulfosalt minerals. The $\chi^{\left( 3 \right)}$ value of abramovite is almost 2.8 times higher than that of the widely used anisotropic nonlinear 2D material of BP, and it is similar to the values of other synthetic nonlinear 2D materials such as GeSe and GeAs. It is also noted that the $\chi_{11} /\chi_{22}$ anisotropy ratio in abramovite is comparable to those in all the other anisotropic vdW materials.

**Table 2:** Comparison of third-order nonlinear susceptibility in abramovite with other anisotropic vdW materials at the fundamental wavelength around 1560 nm.

Material	Crystal system	Space group (bulk)	Thickness (nm)	Band gap (eV)	$\chi^{\left( 3 \right)}$ × 10^–19^ (m^2^ V^-2^)	$\chi_{11} /\chi_{22}$ ratio	References
Abramovite Pb_2_SnInBiS_7_	Triclinic	*P* $\overline{1 }$	26–87	0.94	3.87	~ 1.41	This work
Cylindrite Pb_3_Sn_4_FeSb_2_S_14_	Triclinic	*P* $\overline{1 }$	10–80	0.65	3.06	~ 1.51	^28^
Franckeite (Pb,Sn)_6_Sn_2_FeSb_2_S_14_	Triclinic	*P* $\overline{1 }$	20–100	0.65	1.87	~ 1.23	^56^
Lengenbachite Pb_6_(Ag,Cu)_2_As_4_S_13_	Triclinic	*P* $\overline{1 }$	28–85	1.98	2.18	~ 1.39	^31^
Cannizzarite Pb_46_Bi_54_(S,Se)_127_	Monoclinic	*P*2_1_/*m*	92–173	0.43	0.16	~ 1.75	^32^
Nagyágite [Pb_2_(Pb,Sb)_2_S_4_][(Au,Te)_2_]	Monoclinic	*P*2_1_/*m*	265	< 0.05	0.15	1.09	^55^
Livingstonite HgSb_4_S_8_	Monoclinic	*A*2*/a*	26–82	1.68	0.55	~ 2.04	^63^
Teallite PbSnS_2_	Orthorhombic	*Pnma*	9–61	1.83	3.49	~ 1.16	^64^
Getchellite AsSbS_3_	Monoclinic	*P*2_1_/*a*	17–89	1.74	0.29	~ 1.37	^65^
Gillulyite Tl_2_(As,Sb)_8_S_13_	Monoclinic	*P*2/*n*	34–132	1.71	0.21	~ 1.61	^66^
Gerstleyite Na_2_(Sb,As)_8_S_13_•2H_2_O	Monoclinic	*Cm*	24–123	2.27	0.18	~ 1.83	^67^
Orpiment As_2_S_3_	Monoclinic	*P*2_1_/*n*	13–102	2.5	0.12	~ 1.67	^68^
Black phosphorus	Orthorhombic	*Cmca*	5–20	0.33	1.4	~ 1.8	^59^
ReS_2_	Triclinic	*P* $\overline{1 }$	0.73–10	1.35	35	~ 1.2	^69^
GeSe	Orthorhombic	*Pnma*	10–100	0.87	3.9	1.7–2.1	^54^
GeAs	Monoclinic	*C*2/*m*	10–60	0.65	3.5	2–2.4	^70^
SiP	Orthorhombic	*Cmc*2_1_	10–40	1.69	1.8	~ 1.38	^71^

## Discussion

In summary, we have demonstrated that anisotropic vdW heterostructures of abramovite consisting of alternating Pb_2_BiS_3_-type and SnInS_4_-type 2D material layers can be formed by the mechanical exfoliation of natural sulfosalt mineral abramovite. The thickness of the exfoliated abramovite flake down to 26 nm is achieved, which corresponds to only 22 Q-H layer pairs. With well controlled exfoliation methods such as liquid phase exfoliation, it might be possible to obtain even thinner vdW heterostructures of abramovite. Although the constituent 2D material layers of abramovite crystal are intrinsically isotropic, it is shown by TEM measurements that the forced commensuration between the incommensurate constituent layers results in the out-of-plane rippling which induces the in-plane structural anisotropy in the heterostructure of abramovite. The chemical composition of abramovite is further determined by EDXS studies as Pb_2.39_Sn_1.30_In_0.83_Bi_1.02_(S_6.79_Se_0.21_)_7.00_. The XPS analysis confirms the presence of the positive oxidation states of Pb^2+^, Bi^3+^, Sn^4+^ and In^3+^ in abramovite. The in-plane structural anisotropy of the crystal is unveiled by the optically probed anisotropic phonon vibrational modes using angle-resolved polarized Raman spectroscopy. It is observed that the molecular bonds present in abramovite are analogous to those in the constituent binary sulfides of PbS (galena), Bi_2_S_3_ (bismuthinite), SnS_2_ (berndtite), and In_2_S_3_ (indium sulfide). The anisotropic responses of phonon vibrations in abramovite crystal can be understood in light of the classical Raman tensors. Through polarization-dependent absorption measurements, it is revealed that abramovite thin flakes exhibit strong linear dichroism effect, which is not only important in the context of understanding the origin of optical anisotropy in natural vdW heterostructures and identifying the crystal axes, but also can be harnessed to design future polarization-sensitive photodetectors and transistors. The maximum absorption is obtained when the incident polarization is along the crystal’s *c*-axis, while the minimum absorption is reached with the polarization along the *b*-axis. According to the measured Tauc plot, it is observed that abramovite exhibits an indirect band gap at 0.94 eV and a direct band gap at 1.46 eV. The effect of the structural anisotropy on the nonlinear optical properties of abramovite is further demonstrated by studying the anisotropic THG response from abramovite thin flakes. Through polarization-dependent THG measurements, it is shown that the intensity of the THG emission as the incident linear polarization is along the rippling direction of the crystal’s *c*-axis is almost two times of that as the polarization is along its perpendicular direction of the *b*-axis. The estimated relative magnitudes of the third-order nonlinear susceptibility tensor elements further manifest the intrinsic nature of anisotropic nonlinear optical response in abramovite. Moreover, the third-order nonlinear susceptibility of abramovite is measured as $3.87\times {10}^{-19}$ m^2^ V^−2^, which is almost 2.8 times higher than that of black phosphorus, showing that abramovite exhibits strong third-order optical nonlinearity among the existing natural and synthetic anisotropic vdW materials. These results provide a deeper understanding of the origin of structural, vibrational and optical anisotropy in natural anisotropic vdW heterostructures. Our results not only establish abramovite as a newly discovered natural anisotropic vdW heterostructures with tailored vibrational and optical properties but also can be harnessed for building future linear and nonlinear anisotropic photonic and optoelectronic nanodevices used for optical communication and optical information processing. Further investigation in the effect of the structural anisotropy on the electrical, mechanical and magnetic responses of abramovite will also be valuable for implementing this unique vdW heterostructure into diverse nanodevice applications.

## Methods

### Sample preparation

The quartz substrate is sonicated consecutively in deionized water, acetone, and isopropanol. The bulk natural abramovite mineral (from Kudriavy volcano, Iturup Island, Kuril Islands, Sakhalin Oblast, Russia) is mechanically exfoliated using Nitto tape (SPV 224) to produce the abramovite thin flakes. The exfoliated thin flakes are then transferred directly onto the cleaned quartz substrate by sticking the tape carrying the flakes on the substrate and peeling the tape off fast. For the preparation of the TEM sample, the exfoliated thin flakes are first transferred onto a heat release tape (Revalpha 319Y-4LS). Afterwards the flakes on the heat release tape are transferred onto a copper TEM grid by placing the tape on it and heating at 100 °C for 30 s.

### TEM and EDXS measurement

The TEM imaging and EDXS analysis of the abramovite thin flakes on a copper TEM grid are performed with a JEOL 2100 scanning/transmission electron microscope (S/TEM) operating at 200 keV, with a Bruker Quantax X-Flash detector for EDXS compositional analysis.

### XPS measurement

The XPS spectra from the exfoliated abramovite flakes are collected by the Thermo Scientific Nexsa XPS spectroscopy system. A monochromatic Al K_α_ X-ray source is used and the highest energy resolution of the recorded XPS spectra are less than 0.48 eV. For small spot spectroscopy, an aperture is inserted into the electrostatic lens column to form a virtual probe at the surface.

### Optical setup

The Raman spectrum is collected by focusing a 632.8 nm He–Ne laser beam on the abramovite flake using a 40 × objective lens (NA = 0.6). The linear polarization of the laser beam is defined using a linear polarizer and a rotating half-wave plate. The back-reflected signal is collected by the same objective lens and guided to a spectrometer (Horiba, iHR 550) via a beam splitter. The Rayleigh-scattered laser light is filtered out by putting a Rayleigh rejection filter (Semrock, LP02-633RE-25) before the spectrometer. The parallel polarization component of the Raman spectrum is selected using another linear polarizer before the spectrometer.

For the polarization‐dependent absorption measurements, white light beam from a broadband white light source (Thorlabs, SLS201L, 360–2600 nm) is focused on the abramovite flake by a 100 × objective lens (NA = 0.7). The polarization of the incident white light is defined by placing a linear polarizer before the objective lens. The reflection spectrum is analyzed from the back‐reflected light from the sample which is collected by the same objective lens and directed to the spectrometer using a beam splitter. The transmission spectrum is collected using another 100 × objective lens (NA = 0.7). An iris is used to spatially filter out a small area of the flake in both the cases. The reflectance (*R*) and transmittance (*T*) are obtained by normalizing the measured reflection and transmission spectra with the light source spectra. The absorbance (*A*) spectrum is obtained with the equation *A* = 1 − *R* − *T*. The effect of the absorption in quartz substrate is considered by measuring the absorbance spectrum of quartz substrate in a similar manner. Finally, the absorbance of abramovite flake is calculated by subtracting the measured absorbance of quartz substrate.

For the THG measurement, the pump laser beam at the fundamental wavelength of 1560 nm (Calmar fiber laser, pulse width 90 fs, repetition rate 80 MHz) is focused on the flake using a 40 × objective lens (NA = 0.65). The incident linear polarization of the pump beam is defined by using a linear polarizer and a half-wave plate before the objective lens. The transmitted THG signal from the flake is collected by a 100 × objective lens (NA = 0.7) and analyzed using a spectrometer (Horiba, iHR 550). The transmitted pump laser beam is removed by placing a short pass filter (Thorlabs, FESH 900) before the spectrometer. The polarization state of the collected THG spectrum is selected by using another linear polarizer before the spectrometer.

## Data Availability

The datasets generated during and/or analysed during the current study are available from the corresponding author on reasonable request.
